# Clinical features, etiological spectrum, and outcomes of neurological patients initially presenting with psychiatric symptoms

**DOI:** 10.3389/fneur.2026.1853336

**Published:** 2026-06-22

**Authors:** Chao Chen, Yiya Xu, Zixiong Zhan, Yingchao He, Shifu Xiao, Ting Chen

**Affiliations:** 1 Department of Neurology, Fuzhou University Affiliated Provincial Hospital, Fuzhou, Fujian, China; 2 Department of Neurology, Shengli Clinical Medical College of Fujian Medical University, Fuzhou, China; 3 Department of Geriatric Psychiatry, Shanghai Mental Health Center, Shanghai Jiao Tong University School of Medicine, Shanghai, China; 4 Alzheimer’s Disease and Related Disorders Center, Shanghai Jiao Tong University, Shanghai, China

**Keywords:** autoimmune encephalitis, hyponatremia, neurological disorders, new-onset psychosis, organic causes, psychiatric symptoms, viral encephalitis

## Abstract

**Background:**

Neurological disorders can manifest with psychiatric symptoms as the initial presentation (PSIP), leading to diagnostic challenges and delayed treatment. Data on this patient population remain limited.

**Methods:**

We conducted a retrospective cohort study of patients admitted to a tertiary neurology department between 2019 and 2023 with PSIP. A structured screening protocol using predefined behavioral keywords was applied, followed by independent case review by two neurologists and a psychiatrist to confirm eligibility. Final diagnoses were adjudicated by consensus using contemporary diagnostic criteria. Poor outcome was defined as modified Rankin Scale score >2 at discharge. Multivariable logistic regression was used to identify independent predictors of poor outcome, and a risk score was developed based on these factors.

**Results:**

Of 16,473 screened admissions, 76 patients (mean age 55.8 ± 18.2 years; 42.1% female) met inclusion criteria. Most patients presented with acute-onset (92.1%) non-specific behavioral disturbance (63.2%), and nearly one-third (31.6%) had isolated psychiatric symptoms without any accompanying neurological signs. The leading etiologies were CNS infections (55.3%), predominantly viral encephalitis (40.8%), followed by cerebrovascular diseases (14.5%) and autoimmune encephalitis (13.2%). Poor outcome occurred in 32 patients (42.1%). Independent predictors of poor outcome were hyponatremia on admission (OR, 3.9; 95% CI, 1.3–11.8), viral encephalitis etiology (OR, 3.1; 95% CI, 1.1–8.7), and ICU admission (OR, 4.8; 95% CI, 1.3–17.6). A risk score combining these three factors effectively stratified patients (AUC 0.88; 95% CI, 0.80–0.96), with poor outcome rates ranging from 7.1% (score 0) to 100% (score 3). Subgroup analyses revealed that among viral encephalitis patients, those with PSIP had significantly higher rates of hyponatremia (41.9% vs. 15.2%, *p* = 0.002) and poorer outcomes (58.1% vs. 88.4% good outcome, *p* < 0.001) compared to those without PSIP. Additionally, patients with isolated psychiatric symptoms were younger and had a higher proportion of autoimmune encephalitis (20.8% vs. 9.6%).

**Conclusion:**

PSIP represents a critical clinical situation in which underlying neurological disorders, particularly viral encephalitis and autoimmune encephalitis are common. Hyponatremia serves as a readily available diagnostic clue and independent predictor of poor prognosis. The absence of neurological signs does not exclude serious underlying pathology. However, these findings are derived from a single tertiary center with a modest sample size and require validation in broader populations. Early comprehensive evaluation, including CSF analysis and neuroimaging, is essential to improve outcomes in this diagnostically challenging population.

## Introduction

Psychiatric symptoms are frequently observed in various neurological diseases, such as CNS infections (1, 2), autoimmune encephalitis (3, 4), epilepsy (5), and systemic metabolic disorders (6). In some cases, psychiatric symptoms can be the initial and predominant feature, closely mimicking primary psychiatric disorders such as schizophrenia or mood disorders (7, 8). This diagnostic mimicry often leads to misdiagnosis, and delays in initiating appropriate neurological workup or treatment, which is associated with poorer outcomes.

Despite its clinical importance, the systematic characterization of neurological patients presenting with psychiatric symptoms as initial presentation (PSIP) is limited. Most existing evidence comes from studies focused on specific diseases like autoimmune encephalitis (AE) (3, 4, 9), with a paucity of population-based studies describing the broader etiological spectrum and clinical profile of PSIP patients in a general neurological setting. Here, we conducted a retrospective study of patients admitted to a tertiary neurological department with PSIP. Our primary objectives were to: (1) determine the etiological spectrum of neurological patients presenting with psychiatric symptoms; (2) reveal clinical features of the special patient population in order to prevent delayed diagnosis; (3) explore factors affecting prognosis to guide management and improve outcomes.

## Subjects and methods

### Study design

This retrospective, observational study analyzed data from the medical records of neurological patients who presented with psychiatric symptoms as their initial presenation, and were admitted to the Department of Neurology at Fujian Provincial Hospital of Fuzhou University between January 1, 2019, and December 31, 2023.

The study was approved by the Ethical Review Committee of Fujian Provincial Hospital (K2025-11-006). All procedures performed in this study were conducted in accordance with the ethical standards of the institutional research committee and with the 1964 Helsinki Declaration and its later amendments. The requirement for informed consent was waived due to the retrospective nature of the study by ethical committee.

### Selection of search keywords

As a standardized psychiatric interview was not feasible in this retrospective neurology cohort, therefore, PSIP was defined by expert consensus using predefined descriptive terms. We searched the chief complaint field for a predefined set of Chinese terms commonly used to describe psycho-behavioral disturbances in neurological practice. The terms were established through a multi-step process involving three senior neurologists and one senior psychiatrist.

First, each neurologist independently generated a preliminary list of terms commonly used in the study institution to document psychiatric or behavioral symptoms in the chief complaint field. Second, to ensure comprehensive coverage of terms encountered in real-world practice, we systematically reviewed randomly selected cases with diagnoses known to be associated with psychiatric manifestations. For each year of the study period, 50 cases were randomly selected from seven diagnostic categories: viral encephalitis (10 cases), autoimmune encephalitis (10), central nervous system demyelinating disorders (8), temporal lobe infarction (8), paraneoplastic syndromes (8), metabolic encephalopathy (3), and Creutzfeldt-Jakob disease (3). These categories and case numbers were chosen to represent the spectrum of neurological conditions in which psychiatric symptoms may be the initial presentation. For each selected case, we reviewed the chief complaint to identify any terms not already included in the preliminary lists.

All candidate terms from both sources were discussed in a consensus meeting involving all four reviewers, including three senior neurologist and one senior psychiatrist. Terms were retained if they described symptoms related to cognition, thought, affect, personality, behavior, or speech content, and if all reviewers agreed on their relevance. Terms describing isolated memory impairment or aphasia were excluded, as these represent classic neurological symptoms less specific to the core research question.

Through this process, the following five Chinese terms were selected, with their pinyin equivalents in parentheses: “psychiatric abnormality” (jing shen yi chang), “behavioral disturbance” (xing wei wen luan), “agitation and excitement” (xing fen zao dong), “incoherent speech” (hu yan luan yu), and “slowed responsiveness” (fan ying chi dun). These broad, descriptive terms reflect the language used by neurologists in non-psychiatric settings, who typically document observable behaviors rather than formal psychiatric diagnoses when the underlying etiology is undifferentiated at presentation.

### Patients screening and enrollment

All cases identified through the keyword search underwent a two-step process for screening and enrollment. In the first step, two neurologists independently examined the complete medical records, including the chief complaint and admission history, for each screened patient. The primary objective was to determine whether the chief complaint accurately reflected the patient’s actual presentation and whether psychiatric symptoms were confirmed as the initial and predominant reason for admission. Cases were excluded at this stage if the admission history suggested that the presenting symptoms were primarily attributable to aphasia, dementia, delirium, or altered level of consciousness, rather than to psychiatric symptoms as defined in this study. Disagreements regarding eligibility were resolved through consensus discussion with a senior psychiatrist.

Patients who passed this initial screening were then assessed against predefined inclusion and exclusion criteria for final enrollment. The inclusion criteria were as follows: (1) Age ≥ 14 years; (2) Admitted with new-onset psychiatric symptoms (defined as onset within 4 weeks); (3) Psychiatric symptoms confirmed as the initial and predominant reason for admission. Patients were excluded if: (1) Discrepancy between the chief complaint and other clinical documentation suggesting that the chief complaint may have misrepresented the presenting symptoms; (2) Documented history of a primary psychiatric disorder prior to admission; (3) Present psychiatric symptoms clearly attributable to a known pre-existing systemic medical condition or CNS disorders; (4) Confirmed diagnosis established at another healthcare institution prior to referral to our hospital; (5) Incomplete medical records, defined as missing basic clinical data or insufficient hospitalization length for diagnostic work-up.

### Data collection and definition

Data extracted from EMRs were recorded and defined as follows. Demographic and baseline data included age, sex, marital status, lifestyle factors, and pre-existing comorbidities. Clinical presentation was reviewed by two senior neurologists and one senior psychiatrist. Onset pattern was classified as acute (≤3 days) or subacute (4–28 days). Psychiatric phenotypes were categorized as non-specific behavioral disturbance, mood disturbance, memory impairment, psychotic symptoms, or catatonia, isolated psychiatric presentation was defined as the absence of fever, focal neurological signs, nuchal rigidity, or seizures at admission. Laboratory and imaging data were collected for all patients, with hyponatremia defined as serum sodium <135 mmol/L within 48 h and CSF pleocytosis as white blood cell count >5 × 10⁶/L. Autoantibody testing, NGS, EEG, and brain MRI findings were recorded when performed. Final diagnoses were determined by consensus review of all available evidence by senior neurologists and a senior psychiatrist, applying contemporary diagnostic criteria. The detailed diagnostic criteria for viral encephalitis (10), autoimmune encephalitis including antibody-negative cases (11), acute ischemic stroke (12), Creutzfeldt-Jakob disease (13), neurosyphilis (14), and other neuropsychiatric disorders (15–17) are provided in Supplementary materials. Outcomes were assessed using the modified Rankin Scale (mRS) at discharge: good outcome as mRS 0–2, poor outcome as mRS 3–6 (18). Secondary outcomes included hospital stay, ICU admission, and in-hospital mortality. The viral encephalitis subgroup comprised all patients admitted with viral encephalitis during the study period, identified through diagnosis codes and record review.

All data extraction and classification were performed independently by two attending neurologists, with disagreements resolved by consensus involving a senior neurologist and a senior psychiatrist.

### Risk score development and evaluation

Based on the three independent predictors identified in multivariate logistic regression analysis, we developed a risk score by assigning 1 point to each factor, yielding a total score ranging from 0 to 3. The association between the risk score and poor outcome (mRS > 2) was assessed using the Cochran-Armitage trend test. Discriminatory ability was evaluated by calculating the area under the receiver operating characteristic curve (AUC) with 95% confidence intervals. Sensitivity, specificity, positive predictive value, and negative predictive value were calculated for cutoff points of ≥1 and ≥2. All analyses were exploratory and intended to generate hypotheses for future validation.

## Statistics

Descriptive statistics are presented as mean ± standard deviation for normally distributed continuous variables and as median (interquartile range [IQR]) for non-normally distributed variables. Normality was assessed using the Shapiro–Wilk test. Group comparisons were performed using Student’s t-test for continuous variables and the Chi-square or Fisher’s exact test for categorical variables, as appropriate. To identify factors independently associated with poor outcome (mRS > 2), we performed univariate logistic regression on demographic, clinical, and laboratory variables, followed by multivariate analysis incorporating variables with *p* < 0.10. Results are presented as odds ratios (OR) with 95% confidence intervals (CI). Based on the independent predictors identified in multivariate analysis, we developed a risk score by assigning 1 point to each factor, yielding a total score ranging from 0 to 3. The association between the risk score and poor outcome was assessed using the Cochran-Armitage trend test. Discriminatory ability was evaluated by calculating the area under the receiver operating characteristic curve (AUC) with 95% CI. Sensitivity, specificity, positive predictive value, and negative predictive value were calculated for cutoff points of ≥1 and ≥2. There were no missing data for the primary outcome (mRS at discharge) and for the three key predictor variables in the final multivariate model (hyponatremia on admission, viral encephalitis etiology, and ICU admission). For CSF parameters and MRI findings, missingness was low (<10%) and complete-case analysis was applied. For multivariate analysis, variables with *p* < 0.10 in univariate analysis were entered into the model.

A two-sided *p* value < 0.05 was considered statistically significant. All analyses were conducted using SPSS Statistics version 27.0 (IBM Corp., Armonk, NY, USA).

## Results

### Patient enrollment

**Figure 1:**
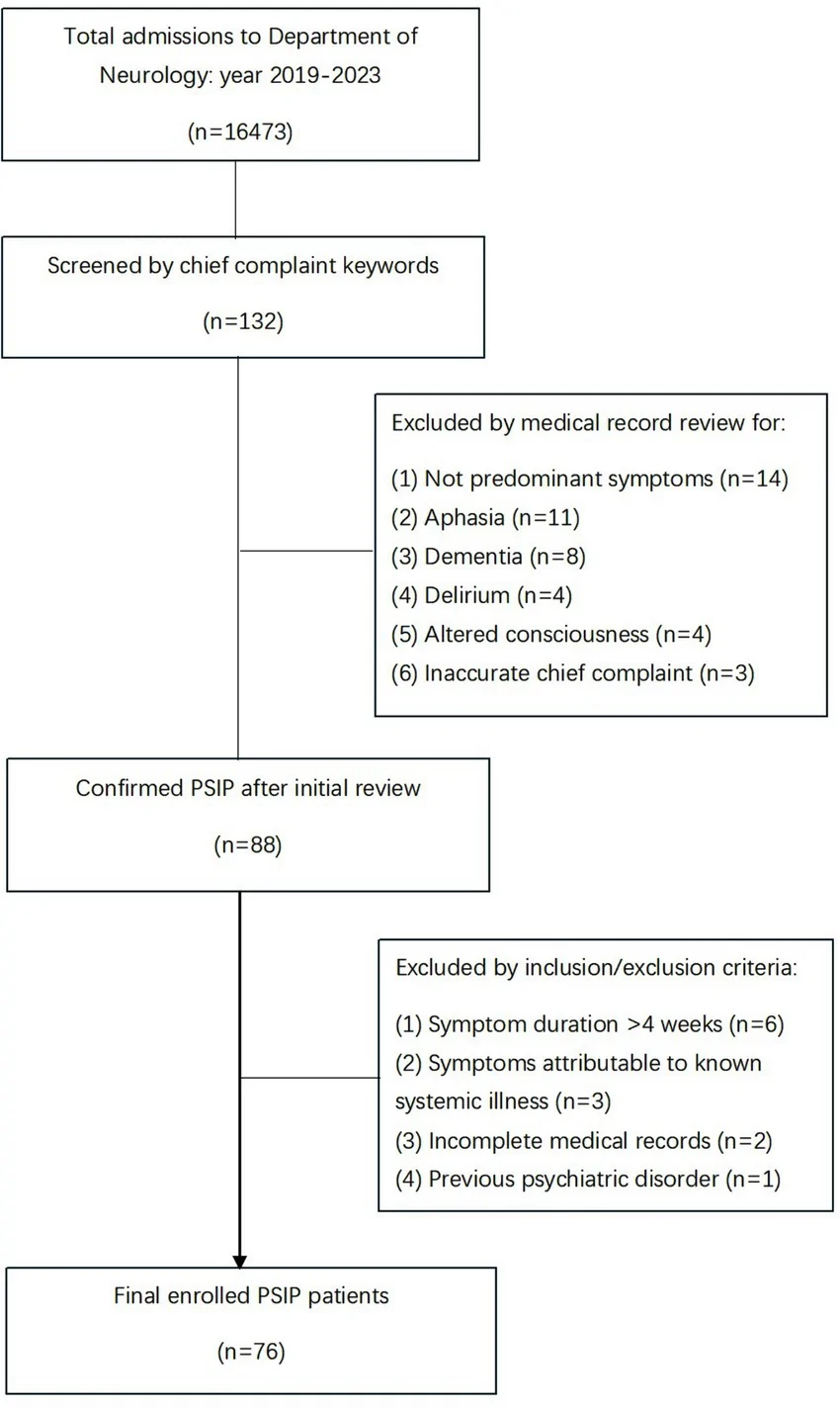
Flowchart of patient screening and enrollment. The flowchart illustrates the two-stage process of patient selection: initial screening by keyword search and medical record review, followed by application of predefined inclusion and exclusion criteria. Numbers of patients excluded at each stage and corresponding reasons are shown in the boxes. PSIP: psychiatric symptoms as initial presentation.

A total of 16,473 patients were admitted to the Department of Neurology between January 1, 2019, and December 31, 2023. Through the keyword-based screening of chief complaints, 132 patients were initially identified as potentially presenting with psychiatric symptoms. After full-text review of admission records by two attending neurologists, 88 patients were confirmed to have psychiatric symptoms as the predominant, initial presenting feature. The remaining 44 were excluded based on the determination that the presenting symptoms fell outside the predefined psychiatric symptom dimensions or did not represent the predominant reason for admission. Following the application of inclusion and exclusion criteria, among the 88 patients identified through screening, 76 patients were enrolled in the final analysis, and 12 were excluded for symptom duration >4 weeks (*n* = 6), symptoms attributable to known systemic illness (*n* = 3), incomplete medical records (*n* = 2), or documented history of primary psychiatric disorder (*n* = 1). The detailed selection process at each stage is presented in Figure 1.

### Patients demographics

The demographic characteristics and baseline comorbidities of the 76 enrolled patients are summarized in Table 1. The mean age was 55.8 ± 18.2 years (range, 14–88 years), with 32 patients (42.1%) being female. The majority of patients were married (62 patients [81.6%]). Cardiovascular risk factors were prevalent, with hypertension (18 patients [23.7%]) and diabetes mellitus (16 patients [21.1%]) being the most common comorbidities, followed by prior stroke (9 patients [11.8%]) and hyperlipidemia (8 patients [10.5%]). A history of malignancy was documented in eight patients (10.5%), and syphilis serology was positive in 5 patients (6.6%). Notably, no patients had a documented history of epilepsy, prior CNS infection, or HIV/AIDS, suggesting that the presenting psychiatric symptoms in this cohort were unlikely to be related to these pre-existing conditions. Smoking and alcohol use were reported in 22 patients (28.9%) and 14 patients (18.4%), respectively.

**Table 1:** Demographics and baseline characteristics of 76 PSIP patients.

Characteristic	Value
Age, years, mean ± SD	55.8 ± 18.2
Age range, years	14–88
Female sex, *n* (%)	32 (42.1)
Marital status, *n* (%)	
Married	62 (81.6)
Single	9 (11.8)
Divorced/widowed	5 (6.6)
Smoking, *n* (%)	22 (28.9)
Alcohol use, *n* (%)	14 (18.4)
Comorbidities, *n* (%)	
Hypertension	18 (23.7)
Diabetes mellitus	16 (21.1)
Prior stroke	9 (11.8)
Hyperlipidemia	8 (10.5)
Malignancy	8 (10.5)
Syphilis (seropositive)	5 (6.6)
CNS trauma	2 (2.6)
Neurodegenerative disease	1 (1.3)
Epilepsy	0
Prior CNS infection	0
HIV/AIDS	0

PSIP, psychiatric symptoms as initial presentation; CNS, central nervous system.

### Clinical features of the patients

The clinical features of the cohort are presented in Table 2. The vast majority of patients (70 patients [92.1%]) experienced an acute onset. The mean time from symptom onset to hospital presentation was 6.8 ± 8.4 days (median, 4 days; [IQR], 2–8 days), reflecting the rapid evolution of symptoms. Through a joint review of medical records conducted by senior psychiatrists and neurologists, the most common psychiatric presentation was non-specific behavioral disturbance (48 patients [63.2%]), encompassing agitation, purposeless movements, mutism, or disorganized behavior in the absence of well-defined psychotic or affective symptoms. This pattern suggests a distinct symptomatic phenotype in a neurological context. Mood disturbance was documented in 15 patients (19.7%), memory impairment in 14 patients (18.4%), and paranoid delusions in 4 patients (5.3%). Hallucinations were notably uncommon and predominantly visual (6 patients [7.9%]) rather than auditory (2 patients [2.6%]). This phenotypic profile, characterized by non-specific behavioral changes rather than classic psychiatric syndromes, may serve as a clinical clue to an underlying organic etiology.

**Table 2:** Clinical characteristics of 76 PSIP patients.

Characteristic	No. (%)
Onset pattern	
Acute (≤3 days)	70 (92.1)
Subacute (4–28 days)	6 (7.9)
Time to presentation, days, median (IQR)	4 (2–8)
Psychiatric manifestations	
Non-specific behavioral disturbance	48 (63.2)
Mood disturbance	15 (19.7)
Memory impairment	14 (18.4)
Paranoid delusions	4 (5.3)
Visual hallucinations	6 (7.9)
Auditory hallucinations	2 (2.6)
Isolated psychiatric presentation*	24 (31.6)
Associated neurological symptoms	
Fever	34 (44.7)
Headache	26 (34.2)
Seizures	18 (23.7)
Altered consciousness	16 (21.1)
Nuchal rigidity	12 (15.8)
Hemiparesis	9 (11.8)
Aphasia	7 (9.2)
Ataxia	5 (6.6)
Sensory disturbance	4 (5.3)
Incontinence	8 (10.5)

*Defined as absence of fever, focal neurological signs, nuchal rigidity, or seizures at admission. PSIP, psychiatric symptoms as initial presentation; IQR, interquartile range.

Among associated neurological symptoms that emerged during hospitalization or were present at admission, fever was the most common (34 patients [44.7%]), followed by headache (26 patients [34.2%]), seizures (18 patients [23.7%]), and altered consciousness (16 patients [21.1%]). Focal neurological deficits, when present, included hemiparesis (9 patients [11.8%]), aphasia (7 patients [9.2%]), ataxia (5 patients [6.6%]), and nuchal rigidity (12 patients [15.8%]). A particularly noteworthy finding was that 24 patients (31.6%) presented with isolated psychiatric symptoms, defined as the complete absence of fever, focal neurological signs, nuchal rigidity, or seizures at the time of admission.

### Auxiliary examination

The pattern of auxiliary examinations reflected routine practice and patient-specific decision-making. All 76 patients (100%) underwent standard-of-care blood tests; 74 (97.4%) had brain MRI. Lumbar puncture was performed in 68 (89.5%), indicating high suspicion for CNS involvement. Application rates were lower for EEG (42 [55.3%]), autoantibody testing (31 [40.8%]), demyelinating antibody testing (12 [15.8%]), and NGS (30 patients [39.5%]), reflecting real-world constraints such as cost, insurance coverage, and selective use based on clinical red flags (19). Routine tests and MRI thus provide generalizable findings, while specialized technical or laboratory investigation offer clinically useful benchmarks when specific diagnoses are suspected, underscoring the study’s aim to identify accessible biomarkers beyond costly, invasive investigations. Detailed results are summarized in Table 3.

**Table 3:** Laboratory and ancillary investigation findings.

Investigation	No./Total (%)
Peripheral blood	
Leukocytosis (>10 × 10^9^/L)	22/76 (28.9)
Hyponatremia (<135 mmol/L)	20/76 (26.3)
Syphilis serology positive	5/76 (6.6)
HIV positive	0/76 (0)
Cerebrospinal fluid	
Elevated opening pressure (>200 mmH₂O)	26/68 (38.2)
Pleocytosis (>5 × 10⁶/L)	40/68 (58.8)
Elevated protein (>45 mg/dL)	39/68 (57.4)
Normal CSF profile	28/68 (41.2)
Autoimmune antibody testing	
Any neural antibody positive	9/31 (29.0)
Anti-NMDAR	5/31 (16.1)
Anti-LGI1	2/31 (6.5)
Anti-GABA-B-R	1/31 (3.2)
Anti-GM1*	1/31 (3.2)
Demyelinating antibody testing	
Anti-MOG positive	1/12 (8.3)
Anti-AQP4 positive	0/12 (0)
CSF NGS	
Pathogen identified	9/30 (30.0)
HSV	6/30 (20.0)
EBV	2/30 (6.7)
VZV	1/30 (3.3)
EEG	
Any abnormality	25/42 (59.5)
Diffuse slowing	22/42 (52.4)
Epileptiform discharges	3/42 (7.1)
Brain MRI	
Any T2/FLAIR hyperintensity	46/74 (62.2)
Cortical	30/74 (40.5)
Limbic	14/74 (18.9)
Basal ganglia	4/74 (5.4)
Thalamic	3/74 (4.1)
Brainstem/cerebellum	4/74 (5.4)
Meningeal enhancement	20/74 (27.0)
Normal MRI	28/74 (37.8)

*Interpreted as accompanying antibody of uncertain significance. CSF, cerebrospinal fluid; NGS, next-generation sequencing; HSV, herpes simplex virus; EBV, Epstein–Barr virusl; VZV, varicella-zoster virus; EEG, electroencephalography; MRI, magnetic resonance imaging; NMDAR, N-methyl-D-aspartate receptor; LGI1, leucine-rich glioma-inactivated 1; GABA-B-R, gamma-aminobutyric acid B receptor; MOG, myelin oligodendrocyte glycoprotein; AQP4, aquaporin-4.

### Peripheral blood findings

Peripheral blood findings revealed leukocytosis (>10 × 10^9^/L) in 22 patients (28.9%), suggesting an underlying inflammatory or infectious process. Hyponatremia (serum Na^+^ < 135 mmol/L) was identified in 20 patients (26.3%) at admission or within the first 48 h, with a mean sodium level of 127.9 ± 4.3 mmol/L in affected individuals. This electrolyte disturbance, often overlooked in psychiatric evaluations, emerged as a frequent and potentially important clue to organic brain disease. Syphilis serology was positive in 5 patients (6.6%), and HIV testing was negative in all patients.

### CSF analysis

Cerebrospinal fluid (CSF) analysis provided direct evidence of CNS involvement in a substantial proportion of those tested. Among the 68 patients who underwent lumbar puncture, elevated opening pressure (>200 mmH₂O) was noted in 26 patients (38.2%), pleocytosis (>5 × 10⁶/L) in 40 patients (58.8%), and elevated protein (>45 mg/dL) in 39 patients (57.4%). However, 28 patients (41.2%) had normal CSF profiles, indicating that a normal lumbar puncture does not exclude an organic etiology, consistent with reports in autoimmune encephalitis where initial CSF may be unremarkable.

### Autoantibodies testing

Autoantibody testing for AE was performed in 31 patients (40.8%), among whom 9 (29.0%) were positive for neural antibodies, including anti-NMDAR (*n* = 5), anti-LGI1 (*n* = 2), anti-GABA-B-R (*n* = 1), and anti-GM1 (*n* = 1). One patients with clinically suspected autoimmune encephalitis but negative antibodies were classified as probable seronegative AE.

Testing for central nervous system demyelinating antibodies (including anti-AQP4, MOG, MBP and GFAP) was performed in 12 patients (15.8%), all of whom had clinical or radiological features raising suspicion for demyelinating disorders. Among these, only 1 patient (8.3%) tested positive for anti-MOG antibodies and was diagnosed with MOG-associated encephalitis.

### CSF next-generation sequencing

Cerebrospinal fluid next-generation sequencing (NGS) was performed in 30 patients (39.5%), typically those with suspected infectious etiologies where conventional testing was unrevealing. NGS identified a causative pathogen in 9 patients (30.0%), including 6 cases of herpes simplex virus (HSV) encephalitis, 2 cases of Epstein–Barr virus (EBV) encephalitis, and 1 case of varicella-zoster virus (VZV) encephalitis.

### Electroencephalography

Electroencephalography (EEG) was performed in 42 patients (55.3%), revealing abnormalities in 25 (59.5%), predominantly diffuse slowing indicative of encephalopathy. Epileptiform discharges were rare, seen in only 3 patients (7.1%), suggesting that while cortical dysfunction is common, overt epileptic activity is not a frequent accompaniment in this population.

### Brain MRI

Brain magnetic resonance imaging (MRI) demonstrated T2/FLAIR hyperintensities in 46 patients (62.2%). Cortical involvement was most common (30 patients [40.5%]), followed by limbic system involvement (14 patients [18.9%]), a pattern suggestive of encephalitis. Meningeal enhancement was present in 20 patients (27.0%), predominantly in infectious etiologies, and served as an important radiological clue to underlying meningitis. Notably, 28 patients (37.8%) had normal brain MRI, reinforcing that a normal study does not rule out CNS pathology.

### Etiological spectrum

The final diagnoses of the 76 enrolled patients revealed a spectrum of underlying neurological disorders which is summerized in Table 4. CNS infections constituted the leading etiology, accounting for 42 patients (55.3%). Among these, viral encephalitis was the most prevalent, diagnosed in 31 patients (40.8%). Pathogen-confirmed cases included HSV encephalitis (*n* = 6), EBV encephalitis (*n* = 2), and VZV encephalitis (*n* = 1), all diagnosed via CSF NGS. The remaining 22 patients with viral encephalitis were classified as unspecified viral encephalitis, diagnosed based on clinical presentation, CSF pleocytosis, and supportive neuroimaging findings without definitive pathogen identification, reflecting the common challenge in clinical practice. Other CNS infections included neurosyphilis (5 patients [6.6%]), bacterial meningitis (4 patients [5.3%]), and tuberculous meningitis (2 patients [2.6%]).

**Table 4:** Final etiological diagnoses of 76 PSIP patients.

Diagnosis	No. (%)
CNS infections	42 (55.3)
Viral encephalitis	31 (40.8)
HSV encephalitis	6 (7.9)
EBV encephalitis	2 (2.6)
VZV encephalitis	1 (1.3)
Unspecified viral encephalitis	22 (28.9)
Neurosyphilis	5 (6.6)
Bacterial meningitis	4 (5.3)
Tuberculous meningitis	2 (2.6)
Autoimmune encephalitis	10 (13.2)
Anti-NMDAR	5 (6.6)
Anti-LGI1	2 (2.6)
Anti-GABA-B-R	1 (1.3)
Anti-GM1 (accompanying)*	1 (1.3)
Probable seronegative	1 (1.3)
Cerebrovascular diseases	11 (14.5)
Ischemic stroke	7 (9.2)
Intracerebral hemorrhage	3 (3.9)
Cerebral venous thrombosis	1 (1.3)
Other diagnoses	13 (17.1)
Non-CNS systemic illness	9 (11.8)
Creutzfeldt-Jakob disease	2 (2.6)
Carcinomatous meningitis	2 (2.6)
Non-organic	2 (2.6)

*Not considered causative, patient classified under viral encephalitis. CNS, central nervous system; HSV, herpes simplex virus; EBV, Epstein–Barr virus; VZV, varicella-zoster virus; NMDAR, N-methyl-D-aspartate receptor; LGI1, leucine-rich glioma-inactivated 1; GABA-B-R, gamma-aminobutyric acid B receptor; GM1, monosialotetrahexosylganglioside.

AE was diagnosed in 10 patients (13.2%). Anti-NMDAR encephalitis was the most common subtype (5 patients [6.6%]), followed by anti-LGI1 (2 patients [2.6%]) and anti-GABA-B-R (1 patient [1.3%]). One patient tested positive for anti-GM1 antibodies. Given that GM1 antibodies are typically associated with peripheral demyelinating neuropathies rather than autoimmune encephalitis (20), this was interpreted as an accompanying antibody of uncertain significance rather than a causative factor. One patient with classic clinical features but negative antibodies was diagnosed as probable seronegative AE.

Cerebrovascular diseases were identified in 11 patients (14.5%), including ischemic stroke (7 patients [9.2%]), intracerebral hemorrhage (3 patients [3.9%]), and cerebral venous thrombosis (1 patient [1.3%]). These cases typically involved strategic locations such as the temporal lobe, thalamus, or limbic structures.

Other diagnoses included Creutzfeldt-Jakob disease (2 patients [2.6%]), carcinomatous meningitis (2 patients [2.6%]), and non-CNS systemic illnesses causing encephalopathy (9 patients [11.8%]), such as metabolic disturbances, nutritional deficiencies, and systemic infections with secondary CNS effects. A final diagnosis of acute transient psychosis was made for 2 patients (2.6%) in the cohort, by joint effort and extensive workup of the senior neurologists and psychiatrist.

### Outcomes and factors associated with poor prognosis

Functional outcomes at discharge were assessed using the modified Rankin Scale (mRS). A good outcome (mRS score 0–2) was achieved in 44 patients (57.9%), indicating functional independence. Poor outcome (mRS score 3–5) occurred in 30 patients (39.5%), reflecting moderate to severe disability, and 2 patients (2.6%) died during hospitalization (mRS score 6). The median length of hospital stay was 16 days (IQR, 11–25 days). Intensive care unit (ICU) admission was required for 14 patients (18.4%), underscoring the severity of illness in a subset of this population.

To identify factors independently associated with poor outcome (mRS score >2), we performed univariate logistic regression on demographic, clinical, and laboratory variables, followed by multivariate analysis incorporating variables with *p* < 0.10. The results are presented in Table 5. In univariate analysis, age >60 years (OR, 2.8; 95% CI, 1.1–7.2; *p* = 0.03), hyponatremia on admission (OR, 4.2; 95% CI, 1.5–12.1; *p* = 0.007), viral encephalitis etiology (OR, 3.5; 95% CI, 1.3–9.2; *p* = 0.01), and ICU admission (OR, 5.8; 95% CI, 1.7–19.8; *p* = 0.005) were significantly associated with poor outcome, with CSF pleocytosis showing a trend toward significance (OR, 2.4; 95% CI, 0.9–6.1; *p* = 0.07). After multivariate adjustment, hyponatremia on admission (OR, 3.9; 95% CI, 1.3–11.8; *p* = 0.02), viral encephalitis etiology (OR, 3.1; 95% CI, 1.1–8.7; *p* = 0.03), and ICU admission (OR, 4.8; 95% CI, 1.3–17.6; *p* = 0.02) remained independently associated with poor outcome. Age >60 years was no longer significant after adjustment (OR, 2.1; 95% CI, 0.7–5.9; *p* = 0.17), suggesting that its effect may be mediated through other factors such as disease severity or comorbidity. These findings highlight hyponatremia as a readily available clinical marker that not only suggests an organic etiology but also portends a worse prognosis, reinforcing the need for aggressive diagnostic and therapeutic intervention in affected patients.

**Table 5:** Univariate and multivariate logistic regression analysis of factors associated with poor outcome (mRS > 2).

Variable	Univariate analysis		Multivariate analysis	
	OR (95% CI)	*p*	OR (95% CI)	*p*
Age >60 years	2.8 (1.1–7.2)	0.03	2.1 (0.7–5.9)	0.17
Female sex	1.2 (0.5–3.0)	0.68	—	—
Acute onset (≤3 days)	1.5 (0.3–8.2)	0.64	—	—
Time to presentation >7 days	1.8 (0.7–4.6)	0.21	—	—
Isolated psychiatric presentation	1.6 (0.6–4.3)	0.33	—	—
Hyponatremia on admission*	4.2 (1.5–12.1)	0.007	3.9 (1.3–11.8)	0.02
Fever	1.3 (0.5–3.2)	0.58	—	—
Seizures	1.8 (0.6–5.2)	0.27	—	—
Focal neurological deficits	1.4 (0.5–4.0)	0.52	—	—
CSF pleocytosis	2.4 (0.9–6.1)	0.07	1.8 (0.6–5.1)	0.26
CSF elevated protein	1.9 (0.8–4.8)	0.16	—	—
MRI abnormality	1.7 (0.7–4.4)	0.25	—	—
Viral encephalitis etiology*	3.5 (1.3–9.2)	0.01	3.1 (1.1–8.7)	0.03
ICU admission*	5.8 (1.7–19.8)	0.005	4.8 (1.3–17.6)	0.02

*indicates *p* < 0.05. Variables with *p* < 0.10 in univariate analysis were included in the multivariate. OR, odds ratio; CI, confidence interval model; ICU, intensive care unit; CSF, cerebrospinal fluid; MRI, magnetic resonance imaging.

### Risk score for predicting poor outcome

Based on the three independent predictors identified in multivariate analysis (hyponatremia on admission, viral encephalitis etiology, and ICU admission), we developed a predictive risk score ranging from 0 to 3 by assigning 1 point to each factor. The distribution of patients across risk score categories and the corresponding rates of poor outcome are presented in Table 6.

**Table 6:** Distribution of risk scores and association with poor outcome.

Risk score	Total patients (*n* = 76)	Patients with poor outcome (mRS >2) (*n* = 32)	Percentage (%)
0	28	2	7.1
1	26	10	38.5
2	18	16	88.9
3	4	4	100

*p* for trend < 0.001 (Cochran-Armitage trend test). mRS: modified Rankin Scale,

Among the 76 patients, 28 (36.8%) had a score of 0, 26 (34.2%) had a score of 1, 18 (23.7%) had a score of 2, and 4 (5.3%) had a score of 3. The rate of poor outcome increased progressively with higher scores: 7.1% (2/28) for a score of 0, 38.5% (10/26) for a score of 1, 88.9% (16/18) for a score of 2, and 100% (4/4) for a score of 3 (P for trend < 0.001). Among the 22 patients with a score of 2 to 3, 20 (90.9%) had a poor outcome, yielding a positive predictive value of 90.9%. Conversely, among the 28 patients with a score of 0, 26 (92.9%) had a good outcome, yielding a negative predictive value of 92.9%.

**Figure 2:**
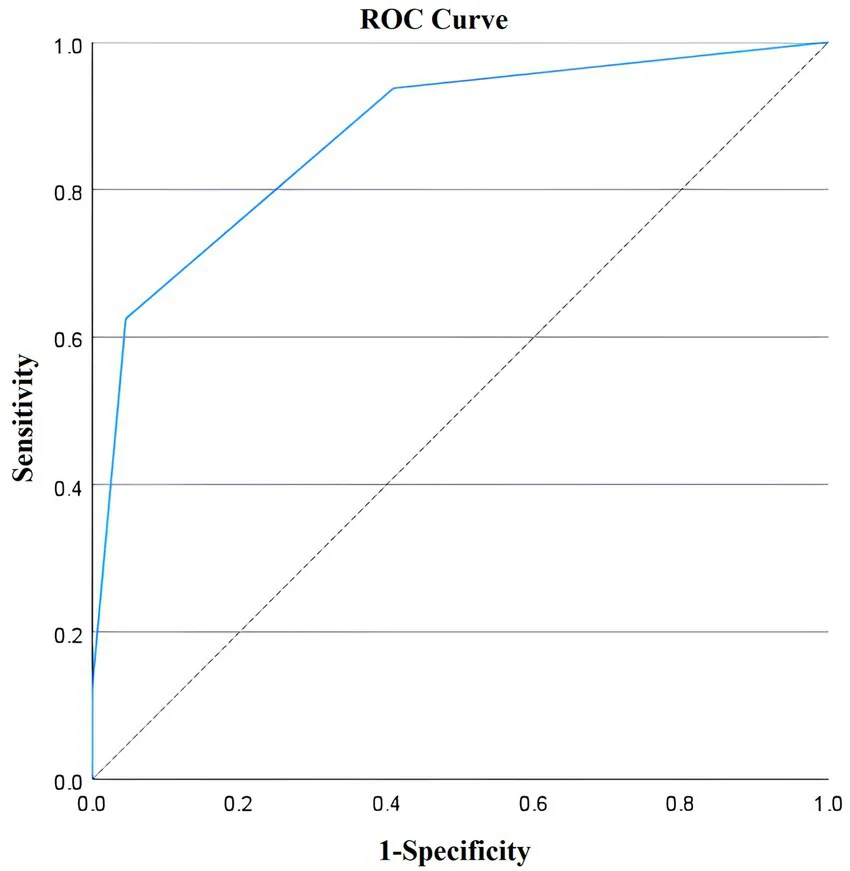
Receiver operating characteristic (ROC) curve of the risk score for predicting poor outcome (mRS > 2) at discharge. The risk score was derived from three independent predictors (hyponatremia on admission, viral encephalitis etiology, and ICU admission). The area under the curve (AUC) was 0.88 (95% confidence interval, 0.80–0.96). The dashed diagonal line represents the line of no discrimination.

The discriminatory ability of the risk score for predicting poor outcome was excellent, with an area under the receiver operating characteristic curve (AUC) of 0.88 (95% CI, 0.80–0.96; *p* < 0.0001) (Figure 2). Using a cutoff of ≥1, the risk score predicted poor outcome with a sensitivity of 93.8%, specificity of 61.4%, positive predictive value of 63.8%, and negative predictive value of 92.9%. Using a cutoff of ≥2, the sensitivity was 68.8%, specificity was 100%, positive predictive value was 100%, and negative predictive value was 80.0%.

## Subgroup analyses

### Viral encephalitis: PSIP vs. non-PSIP

Given that viral encephalitis was the most common etiology in our cohort, we compared patients with psychiatric-predominant presentations to 112 viral encephalitis patients without PSIP admitted during the same study period to determine whether these subgroups differ in clinical features or outcomes. Such differences could inform early recognition and prognostic counseling in this diagnostically challenging population.

As was shown in Table 7, the two groups were comparable in age, sex distribution, time to presentation, and the prevalence of fever, seizures, focal deficits, and routine CSF parameters. However, patients with PSIP had a significantly higher frequency of hyponatremia (41.9% vs. 15.2%; *p* = 0.002) and a markedly lower rate of good outcome (58.1% vs. 88.4%; *p* < 0.001). The absence of significant differences in traditional markers of encephalitis severity suggests that conventional red flags may be less reliable in this subgroup, while hyponatremia emerges as a readily available clue to both diagnosis and prognosis (21). Among the 112 non-PSIP viral encephalitis patients, 22 (19.6%) developed psychiatric symptoms during the illness, confirming that psychiatric involvement is common in viral encephalitis. However, when psychiatric symptoms are the presenting feature, they portend a distinctly worse prognosis.

**Table 7:** Comparison of viral encephalitis patients with and without PSIP.

Characteristic	PSIP (*n* = 31)	Non-PSIP (*n* = 112)	*p*
Age, years, mean ± SD	51.6 ± 18.2	45.2 ± 19.5	0.09
Female sex, *n* (%)	14 (45.2)	43 (38.4)	0.49
Time to presentation, days, median (IQR)	5 (2–10)	4 (2–8)	0.42
Hyponatremia, *n* (%)*	13 (41.9)	17 (15.2)	0.002
CSF pleocytosis, *n* (%)	18 (58.1)	67 (59.8)	0.86
CSF elevated protein, *n* (%)	19 (61.3)	61 (54.5)	0.50
MRI abnormality, *n* (%)	22 (71.0)	72 (64.3)	0.48
ICU admission, *n* (%)	7 (22.6)	16 (14.3)	0.26
Hospital stay, days, median (IQR)	16 (11–24)	15 (10–22)	0.35
Good outcome (mRS 0–2), *n* (%)*	18 (58.1)	99 (88.4)	<0.001

*indicates *p* < 0.05. PSIP, psychiatric symptoms as initial presentation; IQR, interquartile range; CSF, cerebrospinal fluid; MRI, magnetic resonance imaging; mRS, modified Rankin Scale; ICU, intensive care unit.

### Isolated vs. non-isolated psychiatric presentation

Patients presenting with psychiatric symptoms in the absence of any neurological signs pose a particular diagnostic challenge, as they are often initially misdirected to psychiatric services (22). Within our cohort of confirmed organic etiologies, we compared the 24 patients with isolated psychiatric symptoms to the 52 patients with accompanying neurological signs to characterize the clinical and etiological profile of this diagnostically elusive subgroup (Table 8).

**Table 8:** Comparison of patients with isolated vs. non-isolated psychiatric presentation.

Characteristic	Isolated (*n* = 24)	Non-Isolated (*n* = 52)	*p*
Age, years, mean ± SD	51.2 ± 19.3	57.9 ± 17.4	0.14
Female sex, *n* (%)	11 (45.8)	21 (40.4)	0.65
Time to presentation, days, median (IQR)	6 (3–12)	3 (1–7)	**0.046**
Viral encephalitis	8 (33.3)	23 (44.2)	0.37
Autoimmune encephalitis	5 (20.8)	5 (9.6)	0.18
Cerebrovascular	3 (12.5)	8 (15.4)	0.74
CSF pleocytosis, *n* (%)	13/22 (59.1)	32/46 (69.6)	0.40
MRI abnormality, *n* (%)	14/24 (58.3)	33/50 (66.0)	0.52
ICU admission, *n* (%)	5 (20.8)	9 (17.3)	0.71
Good outcome (mRS 0–2), *n* (%)	12 (50.0)	32 (61.5)	0.33

Bold values indicate statistical significance. IQR, interquartile range; CSF, cerebrospinal fluid; MRI, magnetic resonance imaging; mRS, modified Rankin Scale; ICU, intensive care unit.

Patients with isolated presentation were younger (mean age, 51.2 vs. 57.9 years; *p* = 0.14) and had a significantly longer delay from symptom onset to presentation (median, 6 days vs. 3 days; *p* = 0.046). Despite similar rates of CSF abnormalities (pleocytosis: 54.2% vs. 61.5%; *p* = 0.55) and MRI lesions (58.3% vs. 63.5%; *p* = 0.67), the isolated group trended toward worse outcomes (good outcome: 50.0% vs. 61.5%; *p* = 0.33), though this did not reach statistical significance. The etiological distribution differed notably: viral encephalitis was less common in the isolated group (33.3% vs. 44.2%), while autoimmune encephalitis was more frequent (20.8% vs. 9.6%). These findings suggest that among patients with organic brain disease, those presenting with pure psychiatric features are more likely to be younger and to have autoimmune rather than viral etiologies, reinforcing the need for early antibody testing in this population even in the absence of neurological red flags.

## Discussion

This retrospective study provides, to our knowledge, the first systematic characterization of a consecutive cohort of neurological inpatients whose initial and predominant presentation was psychiatric symptoms, who at high risk of misdiagnosis and delayed appropriate care. Our key findings reveal a predominantly organic etiology, with CNS infections, particularly viral encephalitis, being most common. Hyponatremia emerged as a critical red flag, serving as both a diagnostic clue and an independent predictor of poor prognosis, and a simple risk score effectively stratified patient outcomes. Notably, one-third of patients presented with isolated psychiatric symptomsa differeing in demographic characteristics and etiological spectrum. These findings enhance the understanding of neurological disorders initially presenting with psychiatric symptoms and offer practical clinical tools to expedite recognition and guide management.

In the current cohort, nearly all patients presenting with new-onset psychiatric symptoms have an underlying organic etiology, reflecting both the referral patterns of a tertiary general hospital and the initial clinical suspicion guiding admission. Notably, viral encephalitis accounted for the majority of cases (40.8%), whereas autoimmune encephalitis (AE) was identified in only 10 patients (13.2%), following cerebrovascular diseases (14.5%), despite AE being increasingly recognized as a significant cause of new-onset psychosis in the literature (3, 23). Several factors may account for this distribution in the current cohort. First, AE remains a relatively rare condition, and its low absolute incidence inherently limits its detection in a modestly sized cohort. Second, AE often presents with isolated psychiatric symptoms in its early phase, and such patients may be more likely to seek care at psychiatric rather than neurological services (24). Indeed, in our cohort, AE accounted for 20.8% of patients with isolated psychiatric symptoms, compared with only 9.6% of those with accompanying neurological signs, supporting this hypothesis. Third, autoantibody testing was performed in only 40.8% of patients, leaving a substantial proportion of potential AE cases undiagnosed. The high prevalence of viral encephalitis in our cohort likely reflects a underlying epidemiology of our region, where certain viral pathogens are endemic and represent a common cause of encephalitis (25). This distribution is consistent with the expected disease frequency in this population. We recognize that the true prevalence of autoimmune encephalitis in PSIP patients may be higher than reported here, and we strongly encourage future prospective studies with systematic autoantibody testing to better define its frequency. Of note, there are two cases in our cohort diagnosed as acute and acute transient psychosis after comprehensive review, highlighting that even after extensive workup, a small proportion of patients may ultimately be diagnosed with primary psychiatric conditions. Together, these findings underscore the importance of maintaining a low threshold for comprehensive autoantibody testing, particularly in younger patients presenting with isolated psychiatric symptoms, where the likelihood of AE is higher. Failure to pursue such testing may result in missed opportunities for timely immunotherapy, which can substantially alter disease course.

We found that the most frequent phenotype of psychiatric manifestations in our population were atypical symptoms like agitation and disturbed behaviors, which far exceeded well-defined psychiatric symptoms of hallucinations, delusion or manic-depressive mood. Similar results were seen in previous studies of AE, which though recognized only relatively recently, has been established as one of the most common neurological causes of acute new-onset psychiatric symptoms (3, 26). A recent study of 505 cases of anti-NMDA receptor encephalitis (NMDAR-AE) found that behavioral disturbance (68%) was the most common symptom category, followed by psychosis (67%), mood disorder (47%), catatonia (30%) and sleep disturbance (21%) (24). In another review study enrolling 544 cases of NMDAR-AE, Sakis et al., reported that 77% of the cases presented with psychiatric symptoms initially, and agitation was the most frequent manifestation (59%), as compared with psychotic symptoms (54%) (26). Similar phenotypic features of psychiatric manifestation were reported by single case reports or case series of viral encephalitis, CNS demyelinating disorders (27, 28), subacute sclerosing panencephalitis (SSE) (29), or encephalopathy caused by systemic illness (30). While our observation aligns with these reports about the phenotypic characteristics of the studied patients, it is important to acknowledge that the descriptions of the current study were drawn from medical records authored by neurologists rather than psychiatrists. Differences in documentation practices between specialties may have influenced the classification of psychiatric presentations. Nevertheless, this pattern itself carries clinical relevance that when such non-specific behavioral changes, particularly in the context of acute onset are documented, it often reflects a clinical suspicion of an underlying organic etiology, reinforcing the importance of pursuing a neurological workup even in the absence of classic psychiatric syndromes.

Incorporation of peripheral blood test results in early diagnosis offers an easy and minimally invasive way to identify clues to underlying organic causes. A notable peripheral blood-based red flag in our study was hyponatremia, occurring in over one-third of the cohort. Hyponatremia may directly contribute to psychiatric symptoms through cerebral edema or synaptic dysfunction (31, 32), or more commonly, coexist with neurological disorders that cause it via mechanisms such as the syndrome of inappropriate antidiuretic hormone secretion (SIADH) or cerebral salt-wasting syndrome (CSWS) (33, 34). Its association with various neurological conditions, including tubercular meningitis (33), infectious or autoimmune encephalitis (35, 36), subarachnoid hemorrhage (37), CNS neoplasms (38), and demyelinating disorders (39), is well documented. In viral encephalitis, hyponatremia has been shown to predict HSV-1 infection (36), and in anti-LGI1 encephalitis, it correlates with hypothalamic dysfunction (35). Alternatively, hyponatremia may also result from poor oral intake due to altered consciousness or dysphagia. Importantly, our multivariate analysis found its prognostic value to be independent of other severity markers (e.g., ICU admission), suggesting it reflects a specific pathophysiological process, likely involving hypothalamic–pituitary axis dysfunction rather than merely serving as a proxy for overall illness severity. Clinically, the presence of hyponatremia in a patient with acute psychosis should prompt immediate CSF analysis and neuroimaging, avoiding prolonged psychiatric evaluation. Nevertheless, it should be noted that other potential causes of hyponatremia such as medications, comorbidities and hydration status were not fully controlled in the current study due to inherent limitations of retrospective real-world data, prospective studies are needed to further validate this finding.

CSF abnormality can provide direct evidence of underlying neurological disorders. Most of the patients in our cohort undertook CSF analysis, however, nearly half showed no pleocytosis or elevated protein, indicating that a normal CSF profile does not exclude organic illness. This is consistent with reports on AE, where initial CSF findings may be unremarkable (40). Nevertheless, abnormal CSF findings strongly argue against a primary psychiatric disorder, reinforcing the essential role of lumbar puncture even in the absence of clear neurological signs.

The brain MRI findings in our cohort underscore the critical role of neuroimaging in the diagnostic workup of new-onset psychosis, even in the absence of focal neurological signs. While a substantial proportion of patients (60.0%) exhibited cortical T2/FLAIR hyperintensities, a common but non-specific finding associated with a broad range of encephalitic processes, the detection of meningeal enhancement in 28.0% of cases is a particularly significant radiological clue. This finding is frequently overlooked in initial assessments of psychiatric presentations but is a powerful indicator of underlying infectious, autoimmune, or neoplastic meningitis, conditions that are treatable yet carry high morbidity if missed. Its presence should mandate immediate lumbar puncture and aggressive diagnostic pursuit.

The subgroup of viral encephalitis patients with PSIP had a significantly worse prognosis (58.1% good outcome) compared to those without PSIP (88.4%). We believe this difference is largely due to diagnostic delay. Mistakenly attributing symptoms to a primary psychiatric disorder likely delayed lumbar puncture and initiation of antiviral or immunomodulatory therapy. This matters because delayed treatment is known to worsen outcomes in both viral encephalitis where starting acyclovir beyond 48 h is linked to higher mortality and poorer recovery (2, 41), and autoimmune encephalitis, where delayed immunotherapy is associated with worse long-term recovery (40).

CSF analysis plays a critical role in this workup. While nearly half of our patients who underwent lumbar puncture had normal CSF, this does not rule out an organic cause, consistent with reports that autoimmune encephalitis can present with unremarkable initial CSF (40). Conversely, when CSF is abnormal, it provides strong evidence against a primary psychiatric disorder and underscores the value of lumbar puncture even in patients without clear neurological signs.

We identified three independent predictors of poor outcome, including hyponatremia, viral encephalitis etiology, and ICU admission, based on which a simple risk score was developed to help clinicians identify high-risk patients who may benefit from more intensive monitoring and structured follow-up. The main strength of this score is its simplicity, relying on three readily available clinical variables that can be assessed at admission without specialized equipment or laboratory testing. This ease of use makes it potentially applicable in a wide range of clinical settings, including emergency departments and primary care. However, several limitations must be noted. The score is based on a limited set of variables and does not incorporate other potentially important prognostic factors such as age, imaging findings, or CSF parameters (21). Its simplicity, while a strength, may also compromise its discriminative ability in more complex cases. Furthermore, the score was developed from a single-center cohort without external validation in an independent population, which limits its generalizability and clinical applicability at this stage. Future studies should validate this risk score in larger, prospective cohorts and explore whether incorporating additional biomarkers, such as serum neurofilament light chain (NfL) or electroencephalography (EEG) findings that could enhance its predictive performance, and include long-term follow-up (e.g., 6 or 12 months post-discharge) to better understand the trajectory of recovery and identify factors associated with late outcomes. If validated, the tool may help guide early risk stratification and clinical decision-making.

## Limitations

Several limitations must be acknowledged. First, as a single-center study with a modest sample size, our findings may not be generalizable to other populations with different epidemiological profiles. Moreover, our cohort represents a selected group of patients who were admitted to a neurology department, which inherently introduces referral bias, patients with similar symptoms who are directly admitted to psychiatric services or primary care are not captured. Second, the retrospective design introduces inherent selection bias and relies on the completeness and accuracy of medical records. Third, the limited use of CSF autoantibody and NGS testing likely resulted in underestimation of autoimmune encephalitis and specific viral etiologies. The higher proportion of AE in the isolated symptom subgroup suggests that this underestimation may be substantial. Furthermore, the high proportion of viral encephalitis may be influenced by referral patterns, as patients with suspected CNS infections are more likely to be referred to neurology rather than psychiatric services, potentially overrepresenting this etiology in our cohort. Fourth, the small sample size may lead to overfitting of the risk score and imprecise estimates. The risk score was derived without internal validation (e.g., bootstrapping or cross-validation) or calibration assessment. Therefore, it is strictly exploratory and should not be used for clinical decision-making until validated in independent prospective cohorts. Finally, outcomes were only assessed at discharge, precluding analysis of long-term functional status.

## Conclusion

In patients with acute-onset, atypical psychiatric symptoms, even in the absence of neurological signs, an underlying organic CNS disorder should be carefully ruled out. Viral encephalitis is among the most common causes of new-onset psychosis, while autoimmune encephalitis may be more frequent in younger patients with isolated psychiatric symptoms. Hyponatremia serves as a key clinical clue predicting poor prognosis. These conclusions are preliminary and restricted to a tertiary neurology cohort; external validation in broader, more heterogeneous populations, including psychiatric and primary care settings, is required before generalization. The proposed risk score should be viewed as a hypothesis-generating tool, and clinical application awaits independent validation. A thorough diagnostic workup is essential to improve outcomes in this diagnostically challenging population of patients presenting with new-onset psychiatric symptoms.

## Data Availability

The raw data supporting the conclusions of this article will be made available by the authors, without undue reservation.

## Glossary

PSIP: psychiatric symptoms as initial presentation
CNS: central nervous system
CSF: cerebrospinal fluid
MRI: magnetic resonance imaging
mRS: modified Rankin Scale
ICU: intensive care unit
OR: odds ratio
CI: confidence interval
AUC: area under the curve
IQR: interquartile range
AE: autoimmune encephalitis
HSV: herpes simplex virus
EBV: Epstein–Barr virus
VZV: varicella-zoster virus
NGS: next-generation sequencing
EEG: electroencephalography
NMDAR: N-methyl-D-aspartate receptor
LGI1: leucine-rich glioma-inactivated 1
GABA-B-R: gamma-aminobutyric acid B receptor
MOG: myelin oligodendrocyte glycoprotein
AQP4: aquaporin-4
GM1: monosialotetrahexosylganglioside
SIADH: syndrome of inappropriate antidiuretic hormone secretion
CSWS: cerebral salt-wasting syndrome
NfL: neurofilament light chain
