# Correction: Clinical features, etiological spectrum, and outcomes of neurological patients initially presenting with psychiatric symptoms

**DOI:** 10.3389/fneur.2026.1946833

**Published:** 2026-08-13

**Authors:** Chao Chen, Yiya Xu, Zixiong Zhan, Yingchao He, Shifu Xiao, Ting Chen

**Affiliations:** 1 Department of Neurology, Fuzhou University Affiliated Provincial Hospital, Fuzhou, Fujian, China; 2 Department of Neurology, Shengli Clinical Medical College of Fujian Medical University, Fuzhou, China; 3 Department of Geriatric Psychiatry, Shanghai Mental Health Center, Shanghai Jiao Tong University School of Medicine, Shanghai, China; 4 Alzheimer's Disease and Related Disorders Center, Shanghai Jiao Tong University, Shanghai, China

**Keywords:** autoimmune encephalitis, hyponatremia, neurological disorders, new-onset psychosis, organic causes, psychiatric symptoms, viral encephalitis

In the published article, an incorrect number was provided for Joint Funds for the Innovation of Science and Technology, Fujian Province (Grant No: 2024Y9602). The correct number is [2024Y9002].

The original version of this article has been updated.

