# Supplementary material for: Clinical features, etiological spectrum, and outcomes of neurological patients initially presenting with psychiatric symptoms
Source: Front Neurol. 2026 Jun 22;17:1853336. doi: 10.3389/fneur.2026.1853336 (PMC13333461; doi:10.3389/fneur.2026.1853336)
Supplement: Supplementary file 1 [file Table_1.DOCX]

**zDiagnostic Criteria**

**Viral encephalitis:** Clinical presentation (acute febrile encephalopathy with or without focal signs), CSF pleocytosis (>5×10⁶/L), and supportive neuroimaging (T2/FLAIR hyperintensities), with or without pathogen confirmation by PCR or NGS.

**Autoimmune encephalitis:** Graus diagnostic criteria (Lancet Neurol. 2016). Probable seronegative AE defined by clinical features meeting possible AE criteria, exclusion of alternative diagnoses, and negative antibody testing.

**Acute ischemic stroke**: WHO criteria with neuroimaging confirmation (CT/MRI).

**Creutzfeldt-Jakob disease**: WHO diagnostic criteria (progressive dementia, myoclonus, EEG findings, MRI abnormalities, and/or CSF 14-3-3 protein).

**Neurosyphilis**: CDC criteria (positive serum treponemal test plus CSF abnormalities with or without neurological symptoms).

**Cerebral venous thrombosis**: Neuroimaging diagnosis (MR venography or CT venography).

**Non-CNS systemic illnesses**: Diagnoses based on clinical, laboratory, and imaging evidence without primary CNS pathology.

**Reference**

1. Venkatesan A, Tunkel AR, Bloch KC, et al. Case definitions, diagnostic algorithms, and priorities in encephalitis: consensus statement of the International Encephalitis Consortium. Clin Infect Dis. 2013;57(8):1114-1128.

2. Graus F, Titulaer MJ, Balu R, et al. A clinical approach to diagnosis of autoimmune encephalitis. Lancet Neurol. 2016;15(4):391-404.

3. Sacco RL, Kasner SE, Broderick JP, et al. An updated definition of stroke for the 21st century: a statement for healthcare professionals from the American Heart Association/American Stroke Association. Stroke. 2013;44(7):2064-2089.

4. World Health Organization. Global Surveillance, Diagnosis and Therapy of Human Transmissible Spongiform Encephalopathies: Report of a WHO Consultation. Geneva: World Health Organization; 1998.

5. Workowski KA, Bachmann LH, Chan PA, et al. Sexually transmitted infections treatment guidelines, 2021. MMWR Recomm Rep. 2021;70(4):1-187.

6. World Health Organization. *The ICD-10 Classification of Mental and Behavioural Disorders: Clinical Descriptions and Diagnostic Guidelines*. Geneva: World Health Organization; 1992.
